# Development of an education and self-management intervention for chronic headache – CHESS trial (Chronic Headache Education and Self-management Study)

**DOI:** 10.1186/s10194-019-0980-5

**Published:** 2019-03-18

**Authors:** Shilpa Patel, Rachel Potter, Manjit Matharu, Dawn Carnes, Stephanie J. C. Taylor, Vivien Nichols, Tamar Pincus, Martin Underwood, Harbinder Sandhu, On behalf of the CHESS team, Martin Underwood, Felix Achana, Dawn Carnes, Brendan Davies, Sandra Eldridge, David Ellard, Frances Griffiths, Kirstie Haywood, Siew Wan Hee, Manjit Matharu, Dipesh Mistry, Hema Mistry, Vivien Nichols, Stavros Petrou, Shilpa Patel, Tamar Pincus, Rachel Potter, Harbinder Sandhu, Stephanie Taylor

**Affiliations:** 1 Warwick Clinical Trials Unit, Warwick Medical School, University of Warwick, Coventry, CV4 7AL UK; 2 Headache Group, UCL Institute of Neurology and The National Hospital for Neurology and Neurosurgery, Queen Square, London, WC1N 3BG UK; 3 Centre for Primary Care and Public Health, Blizard Institute Barts and The London School of Medicine and Dentistry, Queen Mary University of London, London, UK; 4 Department of Psychology, Royal Holloway, University of London, Surrey, TW20 0EX UK

**Keywords:** Chronic headache, Tension type headache, Migraine, Self-management, Primary care, Behaviour change, Intervention development

## Abstract

**Background:**

Self-management interventions are well recognised and widely used in chronic conditions. Their application to chronic headaches has been limited and generally of low quality. We describe here our process for developing an evidence based, and theory driven, education and self-management intervention for those living with chronic headache.

**Methods:**

Our intervention was designed using several core information sources; the results of three systematic reviews, qualitative material from those living with chronic headaches, our knowledge from existing self-management interventions; and finally collaborative input from a multidisciplinary team of clinicians, academics, patients, and charity partners. We manualised the intervention and associated training as a package for use in a feasibility study. We made adaptations for its use in a randomised controlled trial.

**Results:**

We piloted the intervention in four groups with a total of 18 participants. Qualitative feedback from 12 participants and five facilitators allowed the intervention to be refined for the main randomised controlled trial. Some of the key changes included shortening of the overall intervention, changes to the originally planned facilitators and spreading the facilitator training over three days rather than two.

We are now testing the final revised intervention in a randomised controlled trial of its clinical and cost effectiveness. The group component of the intervention is delivered over two days with the first day focused on living, understanding and dealing with chronic headaches and the second day exploring how to adapt and take control of one’s life with chronic headaches.

**Conclusion:**

Our pilot work indicates that our intervention is feasible to deliver, and with the relevant changes would be acceptable for use with this population. Our randomised control trial is ongoing. We anticipate publishing final results in 2021.

**Trial registration:**

ISRCTN79708100. Registered 16th December 2015, http://www.isrctn.com/ISRCTN79708100

## Background

Chronic headaches affect 2–3% of the population. Globally migraine, medication overuse headache and tension type headache are, respectively, the second, sixth and 12th leading causes of disability from neurological disorders [1]. Around 4% of UK primary care consultations and 30% of neurology outpatient appointments are due to headache disorders [2–4]. The annual direct treatment cost to the UK National Health Service (NHS) is £1 billion [5]. As headache disorders are most prevalent among the working age population they have a large economic impact. The annual cost of headache disorders in the UK is between £5–7 billion; primarily due to lost production [6, 7].

Managing chronic headaches can be challenging, the focus has tended to be on pharmacological interventions. Pharmacological management has tended to focus on episodic migraine, with few studies focusing on chronic headaches [8]. Only topiramate and OnabotulinumtoxinA have been shown to reduced headache days for those with chronic migraine [9–13]. The impact on quality of life has not been explored in trials and the overall use of other pharmacological strategies is very limited and of poor quality amongst a chronic headache population. The UK National Institute for Health and Care Excellence (NICE) published guidance on headaches in September 2012. Apart from a recommendation to consider a course of acupuncture for people with chronic migraine or chronic tension type headache, the guideline developers did not find suitable evidence to allow recommendations on non-pharmacological treatments for people with chronic headache [14].

There is good evidence for supportive self-management programmes for long-term conditions [15]. Such programmes have been used in a range of chronic conditions [16–18]. These use patient education and behaviour change strategies to encourage those living with chronic conditions to engage and take an active role in managing their own condition and to minimise the impact this condition has on individual’s physical and psychological functioning. Chronic conditions can have a substantial impact on individual’s lives [19, 20] therefore a focus on a biopsychosocial approach taking into account physical, psychological and social factors is appropriate [21]. There has been limited high quality evidence for the use of self-management interventions in the treatment of chronic headaches [22], hence the need for further research.

There is some suggestion of an association between chronic headaches and chronic musculoskeletal pain [23, 24]. A systematic review of the association between headache and low back pain found that the odds ratio for the association ranged from 1.55 to 8.0 in different studies [25]. This association maybe linked to central sensitisation, which may provide a common pathway for chronic headache and other chronic pain syndromes [26, 27]. It is therefore appropriate to draw on the current evidence base from other painful chronic conditions to inform strategies to facilitate effective self-management of chronic headaches.

Building on this evidence we have been funded to develop and test a group self-management support programme for people living with chronic headaches (funded by the NIHR Programme Grants for Applied Research programme - RP-PG-1212-20,018) [28]. We describe here the development and initial evaluation of an education and self-management support intervention for people living with chronic headache. The specific aim of the programme is to enable people with chronic headache to manage their pain better and to improve their quality of life. For our main randomised controlled trial, we are testing the hypothesis that amongst adults with chronic headache, the provision of a self-management support programme in addition to best usual NHS care will help to improve headache related quality of life.

## Methods

The Medical Research Council framework for designing complex interventions, the Person-Based Approach and core theoretical principles from psychological models including Michie’s behaviour change wheel and taxonomy have guided the development of our intervention [29–33]. The MRC framework encourages the development of interventions by drawing on theory and evidence based research. The Person-Based Approach suggests behaviour change intervention should be grounded in a detailed insight of the needs, perspectives and context of individuals who will be the recipients. We have adapted the Person-Based Approach and used it as a structure here. Table 1 provides an overview of our intervention development process.

**Table 1:** An overview of our intervention development process using an adapted version of the Person-Based Approach

Intervention development stage	Outputs	Processes undertaken
Planning	Exploration of evidence base to identify patient needs and challenges	Synthesis of evidence using systemic reviews: a. education and self-management interventions for chronic headache [22] b. lived experiences review [35] c. prognostic factors for chronic headache [36] Qualitative material collected via interviews with people living with chronic headache
Design	Outline of needs and challenges to be addressed to meet overall intervention objectives	Creating an outline of the intervention aims and objectives including the key features and components to achieve the objectives using: 1. Our experience of developing and testing an intervention package for people living with chronic musculoskeletal pain, COping with persistent Pain, Effectiveness Research into Self-management (COPERS study) [50, 51] 2. Input from a multidisciplinary team of clinicians, academics, PPI, and charity partners at a collaborative intervention design meeting 3. Outcomes from a classification development day which aimed to inform the development of a logic model to support the classification of chronic headache disorders. Input into this day came from neurologists, headache specialist general practitioners, headache specialist nurses, and people with chronic headache
Development, evaluation and implementation – acceptability and feasibility	Final intervention package evaluated including manuals and training	Feedback from our PPI members attending the intervention design day. Qualitative interviews with facilitators (nurses and lay) and participants from the feasibility study to help refine the intervention for the main RCT.

Throughout the process of designing this intervention, we have drawn upon the views and opinions of our PPI partners to make sure what is produced would be acceptable and useful for those living with chronic headaches. Our PPI support comes from key members of the leading headache charities, members attending our intervention design day, our lay facilitators (who themselves live with chronic headaches) and a wider PPI reference group who have volunteered to support the overall CHESS study with PPI needs.

The development and initial evaluation of the intervention package was just one component of the CHESS feasibility study. A detailed account of the full feasibility phase are described elsewhere [34].

### Planning phase - systematic reviews

We completed three systematic reviews to help inform the development of the intervention. Full details of the methodology and results of these reviews are reported elsewhere [22, 35, 36]. Here we have provided a summary of the key finding and the influence these had on our intervention design (Table 2).

**Table 2:** Summary of results from reviews and influences on intervention design

Key findings	Influences on intervention design
Style and content review [22] To review the effectiveness of self-management interventions for headaches and highlight the differential components included and delivery methods used	
Inclusion of CBT	The overall intervention is informed by the core principles of cognitive behavioural therapy (CBT). The focus being on unhelpful thinking patterns and the need to recognise such thought processes and look for alternatives that are more helpful. Participants have the opportunity to explore the different types of unhelpful thought patterns and subsequently reflect on the challenges these create and ways to make them more helpful/manageable.
Inclusion of education	The programme is an educational and self-management intervention and therefore includes topics that carry a large educational component. This includes topics such as ‘headache information and mechanisms’ and ‘medication management.’
Inclusion of mindfulness	As part of a taster session, mindfulness is included. Participants are provided with a mindfulness CD for home practice.
Inclusion of relaxation	Relaxation is included as a taster session and participants are provided with a copy of the relaxation CD for home practice.
Group interventions more effective	The intervention is group based, aiming to get between 8 and 10 participants per group.
Face to face and remote delivery did not make much of a difference	As this is a complex intervention with several components, we felt a face-to-face, group intervention with a built in one to one consultation would be the best option based on previous experience from the team in delivering complex interventions.
Homework - no difference in studies offering this and not	We included homework as part of our intervention to enable bedding in of information and discussions from day one and to allow any uncertainties to be clarified on day 2. Participants are encouraged to make use of the relaxation CD and to watch the headache DVD.
Email/telephone support – no difference in studies offering this and not	Telephone follow-up is provided as a means of supporting those who are implementing changes and in particular those who might be withdrawing from medication. The frequency of these calls are individually negotiated between the nurse and the participant.
No indication that delivery by a Psychologist or Psychotherapist was any more or less effective than a nurse or Allied Health Professional (AHP)	The collaborative team carefully considered who should facilitate the delivery of the intervention. Due to the medical aspects of headaches around mechanisms, medication and headache classification a nurse was deemed most appropriate.
Lived experiences review [35] To synthesis the qualitative literature on the lived experience of people with chronic headache disorder	
Headaches act as a driver to increase medication	We have specifically included a session on medication to allow exploration of acute and preventative medication. Focus is also given to the concept of medication overuse headaches and subsequently the opportunity to discuss this during a one to one consultation.
Headaches lead to avoidance in planning	The intervention includes the headache pain cycle and the need to break the cycle. We explore the skills associated with identifying barriers to change and using problem solving and goal setting as a means to engaging in meaningful activity. Participants are encouraged to complete their own goal-setting plan and to bring that to the one to one appointment for discussion.
Headaches encouraged changes in sleep patterns	Sleep management is included as a session to enable participants to understand the link between sleep and thoughts and subsequently look at what is, and is not, recommended for good sleep management.
Headaches a driver to stopping doing things	The headache pain cycle is used to discuss a feeling of being trapped and therefore withdrawing. This is further explored to identify strategies to help break this cycle.
A sense of loss of control	The whole intervention is designed to educate and encourage those with chronic headaches to explore strategies to help them better manage their headaches and improve their quality of life. As part of this journey we explore the concept of control and the implication this can have on headaches.
Prognostic review [36] To identify predictors of prognosis in studies of those with chronic headache	
Depression and anxiety	The intervention includes topics around the link between mood and headaches and the impact this can have. We provide participants with a handout outlining the possible symptoms of depression and advise to seek support from their GP if they are struggling with these. Mindfulness and relaxation are built in as strategies to help manage mood and anxiety.
Medication overuse	This is covered as a topic in the facilitated group sessions and then further discussed during the one to one consultation, if relevant.
Poor sleep	The concept of a balanced and healthy lifestyle is facilitated as a topic during the group sessions. As part of this, sleep and effective sleep management strategies are discussed.
High stress	Sessions on managing stress and anxiety are included. Participants are encouraged to explore the impact of stress and anxiety and subsequently look at strategies for management. Relaxation and mindfulness are introduced as strategies to manage stress and participants are encouraged to practice these at home.
Headache management self-efficacy	The course is designed in inform, empower and build confidence in those with headache to take control and use self-management strategies to help them manage their headaches better.

### Planning phase - qualitative interview study

We conducted qualitative interviews to inform the intervention design. This interview data and the lived experience systematic review both aimed to ensure that the intervention design included a strong patient focus. Migraine Action,^1^ one of the charity partners in CHESS, sent letters on the trial’s behalf to their members within a predefined geographical region for ease of travel (100 members resided in this area). Interviews were face to face in people’s homes and were audio recorded after taking informed consent. Topic guides explored their experiences of what might be helpful, or unhelpful, treatment strategies and where they sought information regarding their headaches. Interviews were transcribed, anonymised and analysed thematically. All data were collected and held in accordance with data protection guidelines.

From the 100 invitation letters sent out, we received 21 responses. Of these responses, five had headaches for < 15 days per month, three had no headaches, three were under the age of 18 years and two were not interested in the study. Of the eight that met our inclusion criteria of headaches on > 15 days per month for at least three months, one had new daily persistent headache and was therefore excluded. We interviewed seven people; the results of these interviews were presented at the intervention development day.

When participants were asked about things that they had tried which were helpful for their headaches they spoke about a vast array of treatments and strategies. These included; belonging to organisations for information and support, seeing approachable and knowledgeable doctors, seeing different therapists (physiotherapists, acupuncturist, counsellor, craniosacral therapist), meditation/relaxation, distraction techniques, being outdoors, having social support from people who understand, having a positive mind-set, Yoga, Pilates and breathing techniques. However, what was helpful to some was often unhelpful to others and many of the things tried were out of the scope of our intervention.

When describing their medication use, interviewees spoke of ‘mixing and matching’, and ‘trial and error’, some medication working at one time and not at another. Side effects were an important feature and two interviewees voiced worries about their medication being rationed. Exact medication use was difficult to ascertain at interview. This was an area for us to explore further in the feasibility study interviews.

We asked participants for their views about what we should consider for inclusion in an education and self-management group intervention. Suggestions included getting peer support (group meetings); developing skills in stress management, relaxation/meditation; learning about triggers, lifestyle factors and medication; having information in one place; having education for others such as family and employers; gaining expert support and finding out about the latest research and advances.

They also gave us some practical points about running a group for people with frequent headache, this included running of the course in a neutral environment so that it was not too medical (such as a hospital setting). They felt small groups were important and there should be regular breaks throughout the day. An option to have groups in the evening was also suggested for those that work or cannot attend during the day.

### Design phase - knowledge from existing intervention

With the associated links between chronic headaches and those with chronic pain, we have drawn upon the knowledge and experience of intervention design and delivery from those involved in the design and running of the COPERS study [37]. Key members of that study are part of the CHESS study team. This was a randomised control trial of a group education and self-management intervention for those living with chronic pain. This was a complex intervention designed using underpinning psychological theory and the guidance of the MRC framework for designing complex interventions [32, 33]. This team conducted two reviews to help inform their intervention design and the results of these reviews have been examined as part of the CHESS intervention development [38, 39]. The COPERS intervention was proven to be acceptable and effective in the medium-term for depression, anxiety, social integration and support, pain acceptance, and self-efficacy in pain management. There are also long-term positive effects for depression, and social integration and support.

The basic structure and content of the COPERS intervention was used to inform the CHESS intervention as was the use of groups so that people could learn from each other and the use of lay facilitators to jointly facilitate with the nurse. Having two facilitators allows for easier management of challenges or difficulties without the need to disrupt the rest of the group. Other experience used to inform the CHESS intervention was holding courses in familiar accessible locations and that clinicians from different disciplines were capable of delivering the intervention.

### Design phase – Theoretical underpinnings

We have drawn upon the core theoretical principles from several psychological theories including, many of which have been used in other self-management interventions; cognitive behavioural theory [40, 41], social cognitive theory [42, 43], acceptance and commitment therapy [44], theory of planned behaviour and reasoned action [45, 46] and the health belief model [47, 48]. to guide the development of the intervention aimed at our specific population. The behaviour change wheel and taxonomy [29–31] have been used to guide our thinking about the sources of behaviour that could be targeted, the rational for each topic area and the most effective strategies for implementing and encourage behaviour change. Table 3 summarises the theoretical underpinnings and the behaviour change techniques that have driven the design and components of the CHESS intervention.

**Table 3:** Theoretical underpinnings and behaviour change rational and techniques for CHESS intervention

Modules	Aims	Theoretical underpinnings	Behaviour change taxonomy
Introduction to the course and each other	To make participants feel comfortable and relaxed and encouraging them to participate by introducing themselves to the group.	Biopsychosocial model, social cognitive theory	Breaking barriers and encouraging self and social awareness. Providing opportunity for change through social support, education and managing expectations
Understanding chronic headaches and acceptance	To increase understanding of chronic headaches and reasons for it and to introduce the concept of acceptance and need for self-management.	Principles of acceptance theory, biopsychosocial model	Information and education to increase capability, awareness and shape knowledge. Emotional regulation to enable acceptance
Mind, body and pain link	To start to introduce the concept that pain and mood are linked and that mood can have an influence on headaches. To explain the pain cycle individuals can get stuck in due to the unhelpful things we do, and explore the strategies that can be used to help break the cycle.	Cognitive behaviour theory, fear avoidance model, biopsychosocial model	Education to help shape knowledge and promote capability. Understanding emotional consequence
Dealing with unhelpful thought patterns	To introduce ideas about unhelpful thoughts, automatic thoughts and error in thinking. To understand the impact of unhelpful thinking and how such thought patterns can keep people in the pain cycle and explore ways to reframe these thoughts.	Cognitive behavioural theory, health beliefs model, Biopsychosocial model	Promotes capability in identification and reframing of thoughts
Summary	To clarify learning from day one and provide a reminder for things to do before day 2.		Provide the opportunity for embedding learning through summary and promotion of watching the DVD, promoting self-monitoring (headache diary), encourage behavioural practice (relaxation)
Reflections	To understand and empathise with the group and ascertain current thoughts.	Social cognitive theory, biopsychosocial model	Social support, feedback and monitoring of behaviour, social comparison through feedback, social reward and positive reinforcement
Back to basics	To get participants to think about future goals and explore these by identifying possible barriers, potential solutions and develop an associated action plan. To learn about the importance of lifestyle change by being aware of triggers.	Theory of planned behaviour and reasoned action, Cognitive behaviour theory, biopsychosocial model	Use of education and strategies to encourage enablement and knowledge acquisition. Use of problem solving, personalised goal setting, and action planning. Reflections on individuals capability, motivation and opportunity for change
Making headaches more manageable	To understand the link between stress, anxiety and headaches, and look for strategies that may help manage this better. To understand the link between sleep, anxiety and headaches to help identify strategies that may help improve sleep quality. To help practice the art of being in the present.	Cognitive behaviour theory, Theory of planned behaviour and reasoned action, biopsychosocial model	Regulation and reducing negative emotions. Education and shaping knowledge through instruction on how to perform relaxation and mindfulness with an embedded in session practice. Incentivisation to engage through provision of material to enable behavioural practice and habit formation
Treatment options	To increase knowledge about medication and use of medication for chronic headaches.	Social cognitive model, biopsychosocial model	Information about health consequence, pharmacological support (regulation), self-monitoring (use of headache diaries)
Communication – explaining your headaches to others	To improve listening and communication skills to aid better relationships. To reflect on consulting behaviour and promote effective communication and constructive consultations.	biopsychosocial model	To help with social integration. To promote effective healthcare utilisation. Monitoring of outcomes from previous experiences, use of planning and problem solving, improving communication skills
Future management	To know what to do when experiencing a setback or a flare up.	Cognitive behaviour theory, Theory of planned behaviour and reasoned action, acceptance and commitment therapy. Biopsychosocial model	Preparation and embedded learning.
Summary	To clarify learning from the two days and introduce the structure of the one to one sessions		Embedding learning
One to one session with nurse	To make a classification of headache type and discuss medication management based on the classification. To also review lifestyle factors and goal setting to enable the participant to engage in behaviour change.	biopsychosocial model	Provision of pharmacological information and support as well as embedded learning. Review of goals including reflection on performance and consequence of change. Self-monitoring of headaches and subsequent health, social and environmental consequence. Social reward and positive reinforcement

Grounded in the evidence base from our review, we built in ‘taster’ sessions around relaxation and mindfulness. The sessions provide participant with the opportunity to participate via instruction on how to engage in relaxation and mindfulness. Participants then have the opportunity to take away material to allow for home practice with the aim to encourage regular practice and habit formation in the long- term.

We have also produced a DVD for participants to share with family and friends. The DVD is aimed to be informative, reiterating the core messages from the course as well as portraying what it is like to live with chronic headaches. This was developed in response to suggestions from PPI members at the intervention design day as well as the qualitative review and interviews that suggested those living with chronic headaches feel others do not understand.

### Design phase - collaborative intervention design meeting

This meeting was attended by clinicians including a neurologist and two general practitioners, directors from two leading headache charities (Migraine Action; National Migraine Centre), three lay people living with chronic headaches, psychologists with expertise in self-management and behaviour change, academics and researchers (18 attendees in total). The multidisciplinary team bring together clinical expertise in the management of chronic conditions including headaches and pain as well as expertise in behaviour change and self-management. We drew on this experience to inform the development of the intervention.

The day comprised of short presentations on the results from the three systematic reviews, main findings from the qualitative interviews, an overall study summary from COPERS [37] and a summary of the main outcomes from a classification day which took place to inform the design of a logic model to allow chronic headaches to be classified. The classification day comprised of facilitated discussions on core questions to help inform the development of a logic model. The outcomes from these small group discussions were discussed in a large plenary session and this information was used to help develop and refine a logic model to be used in the study and intervention process. Full details have been published elsewhere [49].

During the intervention design meeting, presentations followed facilitated discussion about the results, what the tailored education self-management intervention should look like, what ongoing support participants would need and what the control intervention should be. We have had input from the three leading headache charities. We have also had the input from three lay members who attended the intervention design day and were involved in the discussions and decisions. After the meeting, a drafting document was circulated which provided all those that attended the opportunity to feedback. This combined input has helped shape the intervention.

## Results

Here we describe the intervention package we developed for implementation based on this work and how we implemented, evaluated and refined ready for use in our RCT.

The CHESS intervention is embedded in the biopsychosocial model, which acknowledges that long-term conditions have physical, psychological and social implication on individuals and therefore management should focus on a combination of these factors. The overall aim of the course was to encourage and enable those with chronic headache to manage and cope with their pain better, to improve their quality of life despite their headache.

### Feasibility intervention design

We designed an education and self-management intervention delivered and facilitated by a non-headache specialist nurse and lay facilitator (someone who lives with chronic headaches). The intervention was delivered over two full days (10:00–15:00) followed by a one to one consultation with the nurse facilitator (agreed at a time convenient for the nurse and participant, approximately a week after the group sessions, lasting up to two hours). This was then followed by a half-day group follow-up session (10:00–12:30). The course was designed for between 8 and 10 participants.

**Fig. 1:**
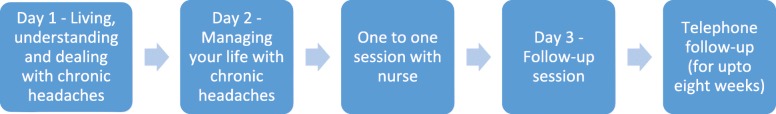
Course structure in the feasibility intervention

Facilitators were recruited through adverts and local contacts. Lay people were recruited through our charity partners. We had three nurses and two lay people interested and available to attend a two day training course aimed to inform them about trial procedures, equip them with facilitation skills for running groups and familiarise them with the content of the intervention. An assessment of learning form was completed by the facilitators following the training to check their understanding, knowledge and confidence in delivering the intervention. Figure 1 shows the structure of the course.

**Fig. 2:**
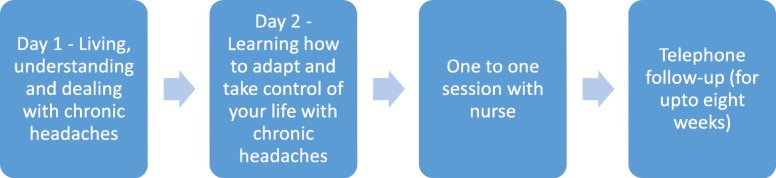
Structure of the CHESS intervention for the randomised controlled trial

The intervention was piloted in four groups in Warwickshire, England with a total of 18 participants. Three of these groups were delivered based on the course structure presented in Fig. 1, the fourth group was delivered using a two day structure (Fig. 2) based on the feedback that had been received. The groups were run in community settings.

We interviewed the five facilitators and 12 of the participants to capture their feedback and experience. We also collected written participant feedback at the end of the course and asked facilitators to provide written reflective logs after each day of facilitation.

Thematic analysis was used to identify common themes across the different components of the intervention. Overall, the results suggested the groups were liked and the material was deemed useful and interesting. Some people had gained new information which was personally useful and others had tried some of the self-management strategies discussed in the group. The opportunity to meet and participate in a group was much appreciated. The feedback from these interviews have enabled the intervention team to streamline the course. Table 4 describes the main changes made following the feedback.

**Table 4:** Summary of the main changes to the intervention following facilitator and participant feedback

Collated feedback summary	Changes to the intervention
Facilitators for the main randomised controlled trial	During the feasibility phase it had been difficult for the lay facilitators to commit to running groups due to their own chronic headaches. This was collaboratively discussed and there was agreement to use an AHP who is registered with an appropriate regulatory body rather than a lay facilitator for the main RCT.
Length of the course	The original 2.5 days was challenging for participants due to work and family commitments. For this reason the half day follow-up was removed. We felt confident that any issues could be raised during the one to one session and subsequent telephone follow-up. The content due to be covered on the follow-up day was merged into day two of the course. This was possible as other topics had been removed and/or revised. The fourth group was run using this two day format.
Timing of the sessions	Originally we had planned to run the two days consecutively, with the follow-up day approximately two weeks later. Two groups were run using this format but participants struggled with some experiencing headaches. The third group was run using a weekly format with one day delivered each week with the follow-up day two weeks after the second group session. In between the second full day and follow-up session, participants had their one to one consultation with the nurse.
Merging of topics related to unhelpful thinking patterns and reframing negative thoughts to positive thoughts	Facilitators found these session difficult to facilitate as they felt disjointed. By merging the sessions it allowed more clarity on the importance of recognising unhelpful thought patterns and subsequently using questions to challenge them and look for more helpful alternatives.
Merging physical activity as a topic into lifestyle factors	Physical activity was included as a separate topic, however it fitted better under the theme of lifestyle factors therefore this topic was integrated into this section.
Removal of session on emerging and complementary treatments	The nurses felt this session was difficult to deliver because of the level of knowledge required and the questions presented to them. For this reason, as well as our understanding that most of these treatments would not be readily available on the NHS we decided to remove this session. We provided participants with a handout detailing the websites of some of the leading headache charities where they could find more information on treatments.
Addition of summary and reminders at the end of day 1	An overall summary table of the content covered was added to allow participants to reflect on the material covered and understand the links between the sessions. We also include some reminders to ensure participants continued to complete the headache diary, watched the DVD and practiced the relaxation where possible.
Changes to some of the session and module names	This was done to improve clarity.
Mindfulness session	This session was broadened to mindfulness and relaxation to give participants the opportunity to understand the differences between the two and have the opportunity to practice these at home.
CHESS intervention training	The training for facilitators in the feasibility phase was delivered over two full consecutive days. The training was delivered by two psychologists, a general practitioner, a neurologist and a nurse by background. From the feedback we found many of the facilitators struggled with the amount of information covered and that they would have liked sometime between the two training sessions. Taking into consideration the feedback, we revised the training for the RCT, it is now delivered over three days, with the first two days as consecutive days covering the group educational and self-management components. This is then followed by a third day, a week later, to cover the training for the one to one session and follow-up phone calls. Further one to one support was provided to each nurse ahead of their first one to one sessions to recap on the logic model and medication advice. In addition, all facilitators are supported individually as and when required by the intervention design team.

Some other more practical issues identified by participants included the need to carefully consider venues for delivering CHESS especially in relation to seating, temperature and lighting. Each venue selected for the CHESS delivery is assessed for suitability by the trial team. The facilitator manual provides a reminder for to facilitators to assess the temperature and lighting and to accommodate needs where possible.

In light of the feedback the structure of the intervention was revised, Fig. 2 shows the structure for the RCT and Table 5 shows the final modules and content of the intervention package.

**Table 5:** Final modules and course content for the CHESS intervention

Day	Modules	Content of sessions
1. Living, understanding and dealing with chronic headaches	1. Introduction to the course and each other	Session 1: Welcome and introductions Session 2: Course overview
	2. Understanding chronic headaches and acceptance	Session 3. Headache information and mechanisms Session 4. Acceptance of chronic headaches
	Taster activity – Relaxation and breathing	
	3. Mind, body and pain link	Session 5. Impact of thoughts, mood and emotions on headaches Session 6. Headache cycle and breaking the cycle
	4. Dealing with unhelpful thought patterns	Session 7. Unhelpful thinking patterns: recognising and finding alternatives
	5. Summary	Session 8: Summary and reminders from day 1
2. Learning how to adapt and take control of your life with chronic headaches	1. Reflections	Session 9. Reflections from Day 1
	2. Back to basics	Session 10. Identifying barriers to change and exploring problem solving and goal setting Session 11. Lifestyle factors and impact on headaches
	3. Making headaches more manageable	Session 12. Managing stress and anxiety Session 13. Managing sleep better Session 14. Mindfulness and relaxation for headaches
	Taster activity – Mindfulness practice	
	5. Treatment options	Session 15. Medication management
	6. Communication – explaining your headaches to others	Session 16. Relationships and communication with family, carers and friends Session 17. Communicating better with Health Professionals
	7. Future management	Session 18. Managing setbacks – what to do when things don’t go to plan
	8. Summary	Session 19. Summary of course
3. One to one session with nurse	Session covers: • Classification assessment with headache diary • Discussion around medication • Lifestyle factors and personalised goal setting.	

## Discussion

The outcomes from our development and pilot work suggest the two-day group intervention followed by a one to one consultation with the nurse and relevant telephone follow-up is feasible and acceptable to a chronic headache population after the recommended changes were applied. Our training package (after the addition of a third day) is sufficient to support facilitators in developing the relevant knowledge base and confidence in facilitating groups.

The development process has mapped onto the MRC framework for developing complex interventions as well as being guided by a Person-Based Approach and core theoretical principles from psychological models and behaviour change theory. We have taken into account the views and input from a range of stakeholders including clinicians, healthcare professionals, academics, patients and charity partners throughout the intervention planning, design and implementation phases.

This work has some potential weaknesses, the number of participants and facilitators was small and we might not have been able to capture the full range of experience in our qualitative work. However, our experience was that no new themes were emerging once interviews were complete. We have not evaluated the revised package before implementing this in the main trial; although the opportunity exists to make minor adjustments following the internal pilot. It is disappointing that we were not able to include lay facilitators but our experience was, that for this study, this was simply not practical.

The PPI element has been an important part of the intervention development process and a particular strength of this work. Our PPI partners have guided us to develop something that is acceptable and relevant for this clinical population. Our early work and the involvement of our PPI partners has allowed us to identify the needs of this population, explore the opportunity to map existing strategies from other interventions, identify any barriers and facilitators to change and subsequently identify appropriate behaviour change techniques to enable implementation of more desired behaviours for the long-term.

The process of developing the intervention to provide a manualised package for use in our definitive randomised controlled trial has been a lengthy process. Despite this, we have produced an intervention that is grounded in the needs of people living with chronic headaches as well as the theory and evidence base.

As part of the intervention development we have also considered quality assurance. For the main RCT the quality, accuracy and approach to delivery are observed and facilitators are provide with feedback. Observations are conducted by members of the study team who are familiar with the intervention. In addition to this, we encourage personal reflection from facilitators via email recording their thoughts and feelings about the sessions, noting things that went well and where things could have gone better. These emails help the intervention design team best support facilitators.

## Conclusion

The CHESS education and self-management intervention is currently being tested in a randomised controlled trial aiming to look at the clinical and cost-effectiveness. We anticipate publishing final results in 2021.

## Abbreviations

AHP: Allied Health Professional
CBT: Cognitive Behavioural Therapy
CHESS: Chronic Headache Education and Self-management Study
COPERS: COping with persistent Pain, Effectiveness Research into Self-management
GP: General Practitioner
MRC: Medical Research Council
NHS: National Health Service
NICE: National Institute for Health and Care Excellence
PPI: Patient and Public Involvement
RCT: Randomised Controlled Trial

## Footnotes

1 In 2018 Migraine Action merged with the Migraine Trust - https://www.migrainetrust.org/

## References

[1] Global, regional, and national burden of neurological disorders during 1990–2015 (2017). a systematic analysis for the Global Burden of Disease Study 2015. Lancet Neurol.

[2] Gahir KK, Larner AJ (2006). Primary headache disorder in the emergency department: perspective from a general neurology outpatient clinic. Emerg Med J.

[3] Hopkins A, Menken M, DeFriese G (1989). A record of patient encounters in neurological practice in the United Kingdom. J Neurol Neurosurg Psychiatry.

[4] Latinovic R, Gulliford M, Ridsdale L (2006). Headache and migraine in primary care: consultation, prescription, and referral rates in a large population. J Neurol Neurosurg Psychiatry.

[5] Ridsdale L, Clark LV, Dowson AJ, Goldstein LH, Jenkins L, McCrone P (2007). How do patients referred to neurologists for headache differ from those managed in primary care?. Br J Gen Pract.

[6] Steiner T (2010) The economic cost of migraine and other headache disorders in the UK: a report of the all-party parliamentary group on primary headache disorders (APPGPHD). House of Commons, London

[7] Chandler JSH, Giles L, Shoesmith D (2018) The Work Foundation: Society’s headache - The socioeconomic impact of migraine. The Work Foundation, London

[8] Jackson JL, Cogbill E, Santana-Davila R, Eldredge C, Collier W, Gradall A (2015). A comparative effectiveness meta-analysis of drugs for the prophylaxis of migraine headache. PLoS One.

[9] Dodick DW, Turkel CC, DeGryse RE, Aurora SK, Silberstein SD, Lipton RB (2010). OnabotulinumtoxinA for treatment of chronic migraine: pooled results from the double-blind, randomized, placebo-controlled phases of the PREEMPT clinical program. Headache..

[10] Silberstein SD, Lipton RB, Dodick DW, Freitag FG, Ramadan N, Mathew N (2007). Efficacy and safety of topiramate for the treatment of chronic migraine: a randomized, double-blind, placebo-controlled trial. Headache..

[11] Diener HC, Bussone G, Van Oene JC, Lahaye M, Schwalen S, Goadsby PJ (2007). Topiramate reduces headache days in chronic migraine: a randomized, double-blind, placebo-controlled study. Cephalalgia.

[12] Mei D, Ferraro D, Zelano G, Capuano A, Vollono C, Gabriele C (2006). Topiramate and triptans revert chronic migraine with medication overuse to episodic migraine. Clin Neuropharmacol.

[13] Silvestrini M, Bartolini M, Coccia M, Baruffaldi R, Taffi R, Provinciali L (2003). Topiramate in the treatment of chronic migraine. Cephalalgia.

[14] NICE. Headaches in over 12s: diagnosis and management - Clinical guideline [CG150] 2012.

[15] Taylor SJC, Pinnock H, Epiphaniou E, Pearce G, Parke HL, Schwappach A (2014). Health services and delivery research. A rapid synthesis of the evidence on interventions supporting self-management for people with long-term conditions: PRISMS - practical systematic review of self-management support for long-term conditions.

[16] Deakin T, McShane CE, Cade JE, Williams RD (2005). Group based training for self-management strategies in people with type 2 diabetes mellitus. Cochrane Database Syst Rev.

[17] Pinnock H, Parke HL, Panagioti M, Daines L, Pearce G, Epiphaniou E (2017). Systematic meta-review of supported self-management for asthma: a healthcare perspective. BMC Med.

[18] Zwerink M, Brusse-Keizer M, van der Valk PD, Zielhuis GA, Monninkhof EM, van der Palen J (2014). Self management for patients with chronic obstructive pulmonary disease. Cochrane Database Syst Rev.

[19] Ruiz de Velasco I, Gonzalez N, Etxeberria Y, Garcia-Monco JC (2003). Quality of life in migraine patients: a qualitative study. Cephalalgia.

[20] Cottrell CK, Drew JB, Waller SE, Holroyd KA, Brose JA, O'Donnell FJ (2002). Perceptions and needs of patients with migraine: a focus group study. J Fam Pract.

[21] Duenas M, Ojeda B, Salazar A, Mico JA, Failde I (2016). A review of chronic pain impact on patients, their social environment and the health care system. J Pain Res.

[22] Probyn K, Bowers H, Mistry D, Caldwell F, Underwood M, Patel S (2017). Non-pharmacological self-management for people living with migraine or tension-type headache: a systematic review including analysis of intervention components. BMJ Open.

[23] Fernandez-de-las-Penas C, Hernandez-Barrera V, Alonso-Blanco C, Palacios-Cena D, Carrasco-Garrido P, Jimenez-Sanchez S (2011). Prevalence of neck and low back pain in community-dwelling adults in Spain: a population-based national study. Spine..

[24] Plesh O, Adams SH, Gansky SA (2012). Self-reported comorbid pains in severe headaches or migraines in a US national sample. Headache..

[25] Vivekanantham A, Edwin C, Pincus T, Matharu M, Parsons H, Underwood M. The association between headache and low back pain: A systematic review. 10.1186/s10194-019-1031-y PMC6734435 31307372

[26] Aurora SK, Kulthia A, Barrodale PM (2011). Mechanism of chronic migraine. Curr Pain Headache Rep.

[27] Obermann M, Nebel K, Schumann C, Holle D, Gizewski ER, Maschke M (2009). Gray matter changes related to chronic posttraumatic headache. Neurology..

[28] Patel S, Carnes D, Eldridge S, Ellard DR, Griffiths F, Haywood K et al Usual care and a self-management support programme vs usual care and a relaxation programme for people living chronic headache disorders: a randomised controlled trial protocol (CHESS). Submited to BMC Medical Research Methodology. 10.1136/bmjopen-2019-033520 PMC7200026 32284387

[29] Michie S, Richardson M, Johnston M, Abraham C, Francis J, Hardeman W (2013). The behavior change technique taxonomy (v1) of 93 hierarchically clustered techniques: building an international consensus for the reporting of behavior change interventions. Ann Behav Med.

[30] Michie S, van Stralen MM, West R (2011). The behaviour change wheel: a new method for characterising and designing behaviour change interventions. Implementation Sci.

[31] Michie S, Atkins L, West R (2014). The behaviour change wheel: a guide to developing interventions.

[32] Medical Research Council - A framework for the development and evaluation of RCTs for complex interventions to improve health. London: 2000.

[33] Craig P, Dieppe P, Macintyre S, Michie S, Nazareth I, Petticrew M (2008). Developing and evaluating complex interventions: the new Medical Research Council guidance. BMJ (Clinical research ed).

[34] White K, Potter R, Patel S, Nichols V, Haywood K, Hee SW et al Chronic Headache Education and Self-management Study (CHESS) - a mixed method feasibility study to inform the design of a randomised controlled trial. Accepted in BMC Medical Research Methodology. 10.1186/s12874-019-0672-5 PMC6371558 30744571

[35] Nichols VP, Ellard DR, Griffiths FE, Kamal A, Underwood M, Taylor SJC (2017). The lived experience of chronic headache: a systematic review and synthesis of the qualitative literature. BMJ Open.

[36] Probyn K, Bowers H, Caldwell F, Mistry D, Underwood M, Matharu M (2017). Prognostic factors for chronic headache: a systematic review. Neurology..

[37] Taylor SJC, Carnes D, Homer K, Pincus T, Kahan BC, Hounsome N (2016). Programme Grants for applied research. Improving the self-management of chronic pain: COping with persistent pain, effectiveness research in self-management (COPERS).

[38] Carnes D, Homer KE, Miles CL, Pincus T, Underwood M, Rahman A (2012). Effective delivery styles and content for self-management interventions for chronic musculoskeletal pain: a systematic literature review. Clin J Pain.

[39] Miles CL, Pincus T, Carnes D, Homer KE, Taylor SJ, Bremner SA (2011). Can we identify how programmes aimed at promoting self-management in musculoskeletal pain work and who benefits? A systematic review of sub-group analysis within RCTs. Eur J Pain.

[40] Beck JS (1964). Cognitive therapy: basics and beyond.

[41] Beck JS (1995). Cognitive therapy: the Guildford press.

[42] Bandura A (1977). Self-efficacy: toward a unifying theory of behavioral change. Psychol Rev.

[43] Bandura A (1986). Social foundations of thought and action: a social cognitive theory: Pearson education.

[44] Hayes SCSK, Wilson KG (2004). Acceptance and commitment therapy: an experiential approach to behavior change.

[45] Ajzen I (1991). The theory of planned behavior. Organ Behav Hum Decis Process.

[46] Fishbein MAI (1975). Belief, attitude, intention, and behavior: an introduction to theory and research.

[47] Rosenstock IM (1966). Why people use health services. Milbank Mem Fund Q.

[48] Rosenstock IM, Strecher VJ, Becker MH (1988). Social learning theory and the health belief model. Health Educ Q.

[49] Potter R, Hee SW, Griffiths F, Dodd K, Hoverd E, Underwood (2019). Development and validation of a telephone classification interview for common chronic headache disorders. J Headache Pain.

[50] Carnes D, Homer K, Underwood M, Pincus T, Rahman A, Taylor SJ (2013). Pain management for chronic musculoskeletal conditions: the development of an evidence-based and theory-informed pain self-management course. BMJ Open.

[51] Taylor SJ, Carnes D, Homer K, Kahan BC, Hounsome N, Eldridge S (2016). Novel three-day, community-based, nonpharmacological group intervention for chronic musculoskeletal pain (COPERS): a randomised clinical trial. PLoS Med.

